# Analysis on the change of gut microbiota and metabolome in lung transplant patients

**DOI:** 10.1128/spectrum.03142-23

**Published:** 2024-02-22

**Authors:** Zhichao Hou, Tangjuan Zhang, Zheng Ding, Ting Qian, Peng Wang, Bo Wu, Xue Pan, Xiangnan Li

**Affiliations:** 1 Department of Thoracic Surgery, The First Affiliated Hospital of Zhengzhou University, Zhengzhou, China; 2 Department of Emergency, The First Affiliated Hospital of Zhengzhou University, Zhengzhou, China; 3 Transplant Center, Wuxi People’s Hospital Affiliated to Nanjing Medical University, Wuxi, China; 4 School of Nursing and Health, Zhengzhou University, Zhengzhou, China; University of Brescia, Brescia, Italy

**Keywords:** gut microbiota, metabolome, lung transplantation, transplant rejection

## Abstract

Previous studies have shown that the gut microbiota and its metabolites are associated with the success of organ transplantation. However, the specific changes in the gut microbiota of lung transplant patients remain unclear. Hence, this study aimed to elucidate the interplay between the gut microbiota, metabolome, and lung transplantation outcomes. Using 16S metagenomics sequencing and untargeted metabolic profiling, we conducted a comprehensive analysis of gut microbial and metabolic alterations in lung transplant recipients relative to non-transplant group. Our findings revealed the predominance of *Enterococcus* and *Streptococcus* genera within the lung transplant cohort, accompanied by the significant reduction in *Bacteroides*, *Epulopiscium*, *Faecalibacterium*, and *Prevotella* abundance. In addition, a significant reduction in ATRA (all-trans retinoic acid) levels and suppression of IgA production were observed in lung transplant recipients, which were found to be closely associated with the *Enterococcus* genus. It was speculated that the association might have implications for the prognosis of lung transplant patients. Notably, the differences in gut microbial composition and metabolomic profiles between successful transplant recipients and those experiencing chronic rejection were not statistically significant. These novel insights shed light on the putative implications of the gut microbiota and metabolome in shaping lung transplantation outcomes, and provide a foundation for future investigations and targeted therapeutic interventions.

**IMPORTANCE:**

This study has profound implications for lung transplantation as it uncovers the important role of gut microbiota and metabolome in shaping transplantation outcomes. The identification of dominant bacterial genera, such as *Enterococcus* and *Streptococcus*, within the lung transplant cohort, along with the significant decrease in *Bacteroides*, *Epulopiscium*, *Faecalibacterium*, and *Prevotella* abundance, reveals potential microbial imbalances associated with lung transplantation. In addition, a significant reduction in ATRA (all-trans retinoic acid) levels and suppression of IgA production were observed in lung transplant recipients, which were found to be closely associated with the *Enterococcus* genus. It was speculated that the association might have implications for the prognosis of lung transplant patients. These findings hold immense clinical significance as they lay the groundwork for future research and targeted therapeutic interventions. Understanding the impact of the gut microbiota and metabolome on lung transplantation outcomes offers promising avenues for improving transplantation patient prognosis.

## INTRODUCTION

The human gut microbiota encompasses an immense population of over 100 trillion microorganisms, including bacteria, viruses, protozoa, and fungi (1). Traditional investigations of the gut microbiota predominantly employed culture-based techniques, limiting the detection and characterization of a fraction of the microbial population. Due to recent advancements in technology, such as culture-independent metagenomic sequencing, researchers have revealed a certain correlation between the gut microbiota and the host’s metabolism, immune response, and inflammatory processes (2, 3).

Organ transplantation is a crucial medical intervention that entails surgically replacing a malfunctioning or damaged organ with a healthy one obtained from a donor. This groundbreaking procedure has revolutionized the field of medicine, granting a renewed lease on life to numerous individuals grappling with organ failure (4). Lung transplantation, in particular, represents a complex undertaking that offers hope to those afflicted by severe lung diseases when all other treatment avenues have been exhausted. The process involves the removal of the diseased lung and subsequent implantation of a viable lung procured from a deceased donor (5). Although lung transplantation can substantially enhance patients’ quality of life and chances of survival, it is not devoid of obstacles, such as the potential for rejection and complications following the transplant (6).

Recent studies indicated that the gut microbiota plays an important role in organ transplantation. Cruz et al.’s study showed that heart transplant recipients exhibited elevated levels of *Lactobacillus*, *Enterococcus faecalis* compared to healthy controls; whereas liver transplant recipients experienced a notable decline in the presence of normal intestinal bacterial species (7). Additionally, heart transplant recipients demonstrated a significant increase in the abundance of anaerobic bacterial species *Lachnospiraceae* and *Ruminococcaceae*, known for their butyrate-producing capabilities, as well as higher concentrations of butyrate in their microbiota compared to liver transplant recipients (7). Dudzicz et al.’s clinical trial proved that the use of the probiotic *Lactobacillus plantarum* 299v could significantly reduce the incidence of *Clostridium difficile* infection (CDI) in kidney transplantation, thereby increasing the survival rate of kidney transplant recipients (8).

Despite the considerable body of research examining the influence of gut microbiota modifications in different types of solid organ transplantation, the exploration of gut microbiota changes in the context of lung transplantation has received limited attention. It is reported that gut microbiota actively engages in bidirectional communication with lungs through the gut-lung axis (9). The axis involves intricate interactions between immune cells, cytokines, metabolites, and neural pathways, which collectively contribute to the modulation of immune responses and inflammation in both the gut and the lung (10). Hence, it is plausible to hypothesize that the gut microbiota might exert an impact on the outcome of lung transplantation through gut-lung axis.

In the present study, we combined 16S amplicon metagenomic sequencing and untargeted metabolomic strategies to investigate the changes in the gut microbiota and metabolome of end-stage lung disease patients among no transplant, successful lung transplant, and lung transplant chronic rejection groups. Additionally, this study endeavored to investigate the underlying mechanisms involved in these gut microbiota alterations, with the aim of offering novel insights and potential therapeutic avenues for the management of prospective lung transplant recipients.

## MATERIALS AND METHODS

### Ethics

The present study was approved by the Institutional Ethics Committee of the First Affiliated Hospital of Zhengzhou University (Ethics Approval No. 2019043), including retrospective review, non-interventional design, and data analysis. All patients in the study were anonymized. The study was conducted in accordance with the Declaration of Helsinki of 2000 and the Declaration of Istanbul of 2008. The Institutional Ethics Committee approved the transplant procedures, and the transplanted organs were donated by volunteers. No lung tissue was obtained from executed prisoners. Written informed consent for the transplantation surgery was obtained from the patients and their close relatives. The transplanted organs were donated by volunteers, and their close relatives voluntarily provided written informed consent.

### Study population

All participants were patients with end-stage lung disease from Wuxi Lung Transplantation Center, which has the largest number of cases in China. The current study involved 52 samples from 52 participants, and these samples were categorized into three groups: no transplant group (NT) 20 samples, lung transplant event-free group (EF) 15 samples, and lung transplant chronic rejection group (CR) 17 samples. The lung transplant rejection participants were patients who survived more than 6 months after transplant and were all cases of chronic rejection. Post-transplant rejections were confirmed by a multidisciplinary team, including thoracic surgeons, respiratory physicians, pathologists, and radiologists. According to the results of the most recent biopsy, cases without any symptoms and with negative pathology were considered event-free patients after transplantation. None of the individuals in the study used antimicrobial agents in the month prior to enrollment or had any evidence of systemic bacterial, viral, or fungal infection. The fecal samples were collected over a 2-year period from 2021 to 2022.

### Sampling and DNA extraction

Upon collection, all fecal samples were immediately frozen at −80°C and stored for later use. At the beginning of the experiment, each sample weighing 180–200 mg was measured and transferred to a 2-mL centrifuge tube, which was then placed on ice. DNA extraction from the samples was performed using the repeated bead beating plus column (RBB + C) method. In brief, ASL buffer (1.4 mL) was added to each sample, followed by the addition of sterile zirconia beads (0.4 g in total, consisting of 0.3 g of 0.1-mm beads and 0.1 g of 0.5-mm beads). The samples underwent bead beating (3 minutes, maximum speed) using a Mini-BeadBeater-16 cell disruptor (BioSpec Products, Oklahoma, USA), followed by incubation at 70°C for 15 minutes with thorough shaking every 5 minutes to facilitate lysis. The subsequent steps of the DNA extraction protocol followed the instructions provided by the manufacturer of the QIAamp Stool Mini Kit (Qiagen, Germany) for bacterial DNA extraction.

### 16S amplicon metagenomic sequencing

#### Amplicon generation

The target regions, including 16S V4 (515F-806R), 18S V4 (528F-706R), and ITS1 (ITS5-1737F, ITS2-2043r), were amplified by PCR using specific primers containing barcodes. Each PCR reaction consisted of 30-µL volume, containing 15 µL of Phusion High-Fidelity PCR Master Mix (New England Biolabs, Massachusetts, USA), 0.2 µM of forward and reverse primers, and approximately 10 ng of template DNA. The thermal cycling protocol began with an initial denaturation step at 98°C for 1 minute, followed by 30 cycles of denaturation at 98°C for 10 seconds, annealing at 50°C for 30 seconds, and elongation at 72°C for 60 seconds. Finally, a final extension step was performed at 72°C for 5 minutes.

#### PCR products mixing and purification

The PCR products were subjected to electrophoresis on a 2% agarose gel at a concentration of 2%. The PCR products that passed the quality control were purified using magnetic beads and were quantified using an enzyme-linked assay. The purified PCR products were then mixed in equal amounts based on their concentrations and were thoroughly mixed. Subsequently, the mixed PCR products were analyzed again by electrophoresis on a 2% agarose gel. The desired bands were recovered using the gel recovery kit provided by Qiagen (Hilden, Germany).

#### Library preparation and sequencing

Sequencing libraries were generated using TruSeq DNA PCR-Free Sample Preparation Kit (Illumina, California, USA). The library quality was assessed on the Qubit 2.0 Fluorometer (Thermo Fisher, Massachusetts, USA) and quantitative PCR (qPCR) quantification. At last, the library was sequenced on NovaSeq 6000 System (Illumina, California, USA).

### High-performance liquid chromatography-mass spectrometry

Each fecal sample (100 mg) was mixed with 600 µL of methanol containing 2-chlorophenylalanine at a concentration of 4 ppm and was vortexed for 30 seconds. The resulting homogenates were sonicated at room temperature for 10 minutes. After centrifugation at 4°C and 12,000 rpm for 10 minutes, the supernatants were collected and filtered through a 0.22-µm membrane for subsequent analysis using high-performance liquid chromatography-mass spectrometry (HPLC-MS/MS). Quality control (QC) samples were prepared using the same procedures. HPLC-MS/MS analysis was conducted by Suzhou PANOMIX Biomedical Tech Co., Ltd. (Suzhou, China). Chromatographic separation was achieved using an ACQUITY UPLC HSS T3 column (150 × 2.1 mm, 1.8-µm particle size) maintained at 40°C. Data-dependent acquisition in the MS/MS experiment was performed using high-energy collision-induced dissociation (HCD) scan.

### Bioinformatics analyses

#### 16S rRNA sequencing data processing

We used QIIME2 in a Docker environment to process the microbiome data (11). Raw sequencing data were quality-filtered and denoised based on DADA2 (divisive amplicon denoising algorithm 2) pipeline (12). All amplicon sequence variants (ASVs) were aligned using MAFFT (multiple alignment using fast Fourier transform) (13). In processing 16S rRNA amplicon data, the ASV method has been proven more sensitive and specific than the traditional OTU (operational taxonomic unit) approach, which produces fewer false sequences, and improves the accuracy, comprehensiveness, and repeatability of data analysis (14). Taxonomic assignment was then performed for representative sequences from each ASV using a QIIME2 naive Bayesian classifier trained on Silva 138.1 SSU rRNA database (15, 16). Taxonomic information, including phylum, class, order, family, genus, and species, was obtained to analyze the community composition of each sample at different taxonomic levels.

#### Analysis of alpha diversity

Alpha diversity refers to the diversity within a single community or sample. It provides information about the number of different species (richness) and the distribution of individuals among those species (evenness) (17). QIIME2 and R language (version 4.3.1) were used to perform alpha diversity analysis (11).

#### Analysis of beta diversity

Beta diversity quantifies the differences in species composition between different communities or samples. It allows for the comparison of microbial communities based on their microbial composition (17). QIIME2 and R language (version 4.3.1) were used to perform beta diversity analysis (11).

#### Linear discriminant analysis effect size analysis

Linear discriminant analysis effect size (LEfSe) is a computational tool used to analyze microbiome data and identify biomarkers or discriminative features within the gut microbiota. Linear discriminant analysis (LDA) is a statistical method employed by LEfSe to determine the significance and effect size of these biomarkers (18).

#### Data preprocessing for HPLC-MS/MS raw data

The raw metabolite data were converted to mzXML format using Proteowizard software (19). Subsequently, peak identification, filtration, and alignment were carried out using R package “XCMS" (20).

#### Untargeted metabolomic analysis

The identified metabolites were matched against the Metlin database (metlin.scripps.edu), MoNA database (mona.fiehnlab.ucdavis.edu), and an in-house MS2 database. Data were normalized through square root transformation and auto-scaling (mean-centered and divided by the square root of the standard deviation of each variable). Variable importance in projection (VIP) scores, calculated using orthogonal projection to orthogonal partial-least squares discriminant analysis (OPLS-DA), were used to assess the significance of each variable in differentiating the metabolome. Differential metabolites were selected based on the following criteria in pairwise comparisons between groups: (i) VIP > 1 in the OPLS-DA model, (ii) *P* < 0.05 (including the methods used for testing and *P* value correction). KEGG Pathway enrichment analysis was performed using MetaboAnalyst software (version 5.0) based on KEGG global metabolic network (21).

#### Integrated analysis of microbiome data and metabolome data

We conducted Spearman correlation analysis between differential microbial genera/species and the differential metabolites.

### Statistical analysis

All statistical analyses were conducted by Student’s *t*-test or one-way ANOVA (analysis of variance) with post-hoc Tukey HSD (honestly significant difference) test using R language (version 4.3.1), and a *P* value < 0.05 was considered as a significant difference. Spearman correlation analysis was also performed using R (version 4.3.1), and the *P* values were adjusted using FDR (false discovery rate) correction, and an adjusted *P* value < 0.05 was considered as a significant correlation.

## RESULTS

### Changes to gut microbiota composition in lung transplant recipients

The alpha diversity analysis primarily incorporates two indicators, namely, Simpson’s diversity (D) index in the form of (1 – D) and Shannon index. Simpson’s diversity index represents the probability of randomly selecting two individuals belonging to different species within a target community. A higher value indicates greater diversity, with Simpson’s index sensitive to species evenness and dominant species. On the other hand, Shannon index represents the uncertainty of randomly selecting an individual and determining its species category within the target community. A higher value indicates higher diversity, with Shannon index emphasizing species richness and the contribution of rare species to diversity.

**Fig 1:**
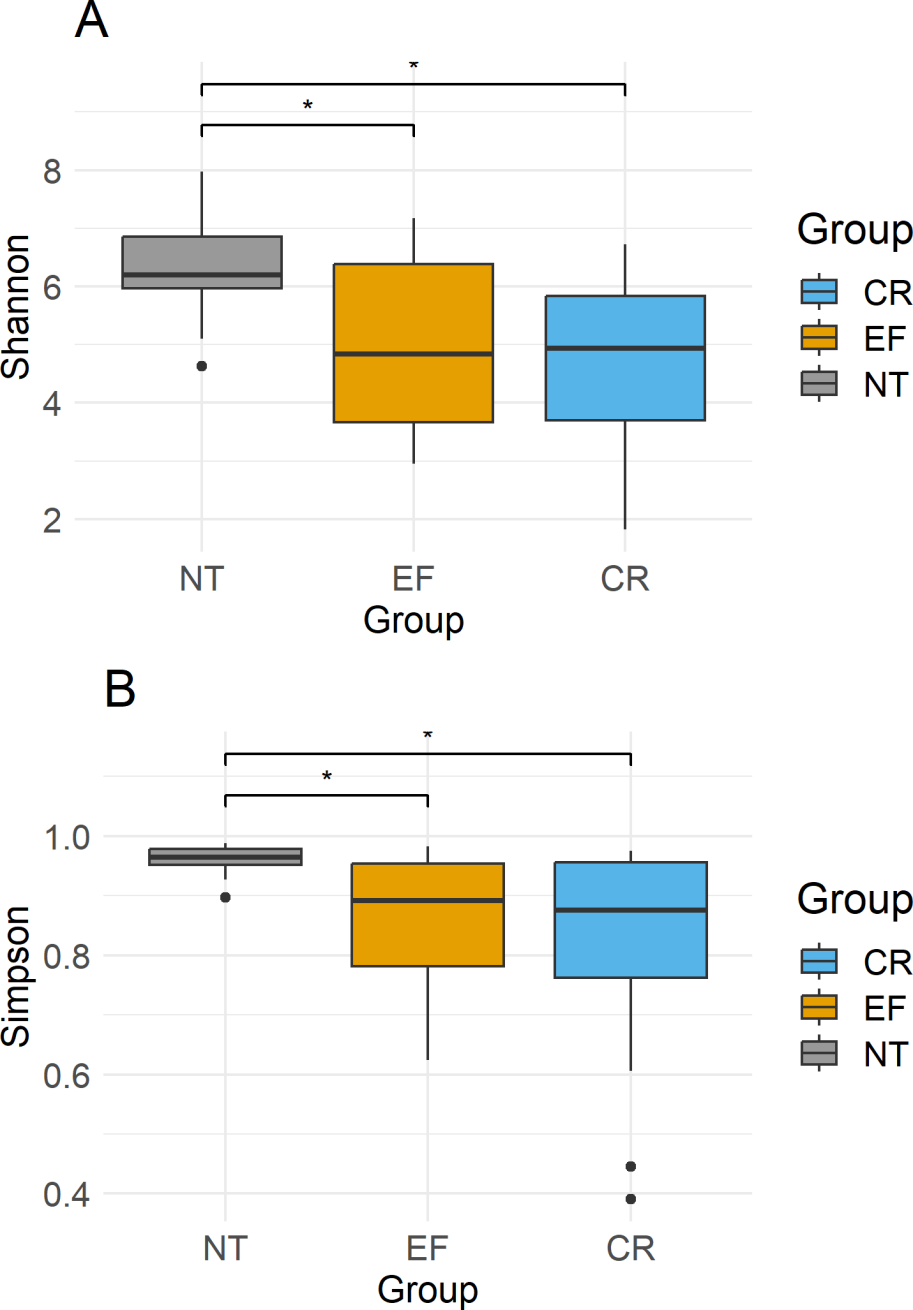
One-way ANOVA for Simpson’s index (**A**) and Shannon index (**B**) among the three groups NT, EF, and CR with post-hoc Tukey HSD. *P value < 0.05, ** <0.01, *** <0.001.

To analyze the distribution of Simpson’s index diversity and Shannon index among different groups, we utilized one-way ANOVA and conducted Tukey’s post-hoc multiple comparisons test. These two indicators revealed significant differences between NT vs EF and NT vs CR (*P* < 0.05), but no significant difference between EF vs CR (*P* > 0.05; Fig. 1).

**Fig 2:**
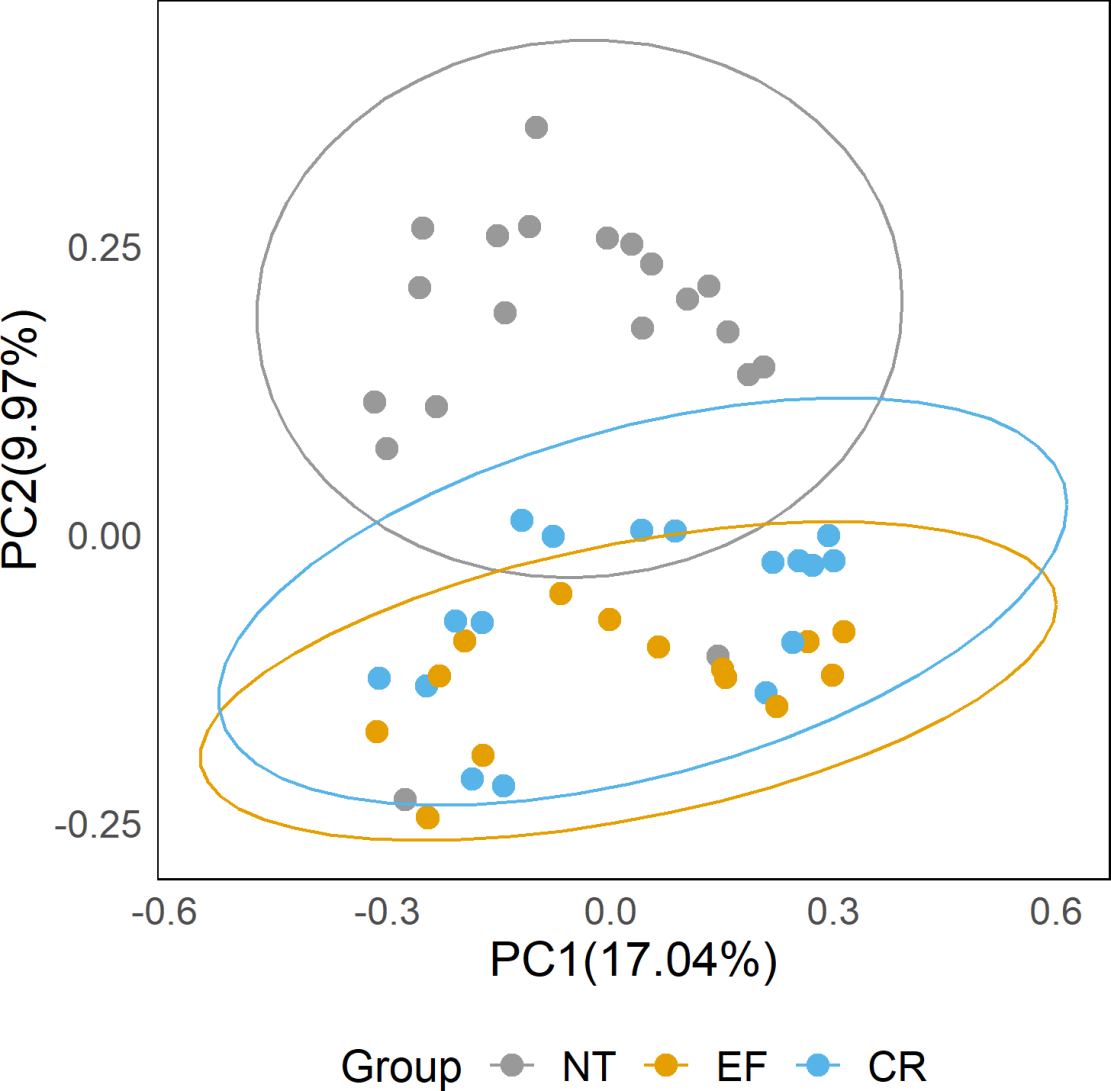
PCoA for the three groups NT, EF, and CR with clustering.

Principal coordinates analysis (PCoA) was performed based on unweighted UniFrac distance. The PCoA with clustering results indicated distinct differences between the NT and the other two groups (EF and CR) with the exception of a few outliers. However, the samples in the EF and CR groups were not clearly separated from each other (Fig. 2).

Adonis (also known as PERMANOVA, permutational multivariate analysis of variance) and Anosim (analysis of similarities) were utilized to further validate the PCoA results. Adonis was used to assess the explanatory power of different grouping factors on sample dissimilarities, while Anosim compared the magnitude of differences between groups and within groups to determine the significance of the grouping. Both Adonis and Anosim results indicated significant differences between the NT group and the other two groups (*P* < 0.001); whereas, the intergroup differences between EF and CR were not significant (*P* > 0.05; Tables 1 and 2).

**TABLE 1:** Adonis analysis for the three groups NT, EF, and CR

Versus groups	Sums of sqs	Mean sqs	F model	*R* ^2^	*P* value
NT-EF	2.12085 (10.88655)	2.12085 (0.32019)	6.62367	0.16305 (0.83695)	<0.001
NT-CR	1.87554 (12.0408)	1.87554 (0.32543)	5.76332	0.13477 (0.86523)	<0.001
EF-CR	0.39077 (11.35238)	0.39077 (0.34401)	1.13594	0.03328 (0.96672)	0.24

**TABLE 2:** Anosim analysis for the three groups NT, EF, and CR

Versus groups	*R* value	*P* value
NT-EF	0.73913	<0.001
NT-CR	0.56156	<0.001
EF-CR	0.02523	0.22

### Differential genus/species abundance

The number of ASVs obtained after species classification annotation is illustrated in Table 3. Some certain ASVs could be annotated at the species (s) level, while the majority of annotations were enriched at the genus (g) level. Therefore, subsequent analyses mainly focused on the genus and species levels.

**TABLE 3:** Number of ASVs obtained after species classification annotation

ASV no.	NT	EF	CR
Phylum	41	44	50
Class	88	110	107
Order	219	216	207
Family	349	320	324
Genus	721	658	621
Species	350	328	334

**Fig 3:**
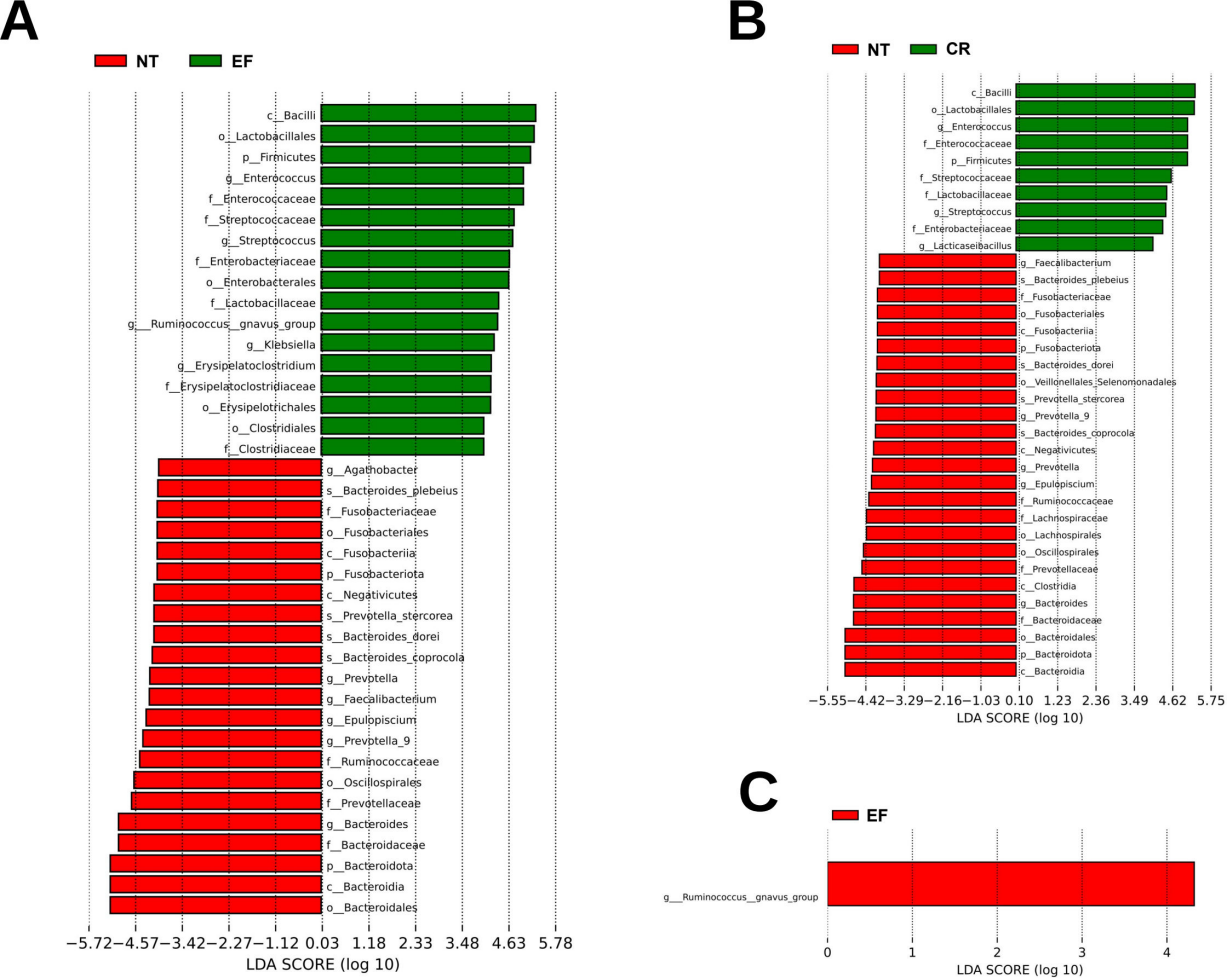
LEfSe analysis for NT vs EF (**A**), NT vs CR (**B**), and EF vs CR (**C**).

The LEfSe analysis was employed to identify the microbial species that exhibited the most significant differences between groups. In the comparison between NT and EF (Fig. 3A), the NT group exhibited a higher abundance of *Bacteroides*, *Epulopiscium*, *Faecalibacterium*, *Prevotella*, *Fusobacteriota*, and *Agathobacter* genera. Specifically, the dominant species in the NT group were *Bacteroides coprocola*, *Bacteroides dorei*, *Bacteroides plebeius*, and *Prevotella stercorea*. The EF group showed a higher abundance of *Erysipelatoclostridium*, *Klebsiella*, *Ruminococcus*, *Enterococcus*, and *Streptococcus* genera.

When comparing the NT and CR groups (Fig. 3B), the NT group displayed a higher abundance of *Bacteroides*, *Epulopiscium*, *Faecalibacterium*, and *Prevotella* genera. The dominant species in the NT group were *Bacteroides coprocola*, *Bacteroides dorei*, *Bacteroides plebeius*, and *Prevotella stercorea*. Conversely, the CR group exhibited a higher abundance of *Lacticaseibacillus*, *Enterococcus*, and *Streptococcus* genera.

In analyzing the EF and CR cohorts (Fig. 3C), only *Ruminococcus gnavus* group was found dominant in the EF group.

### Differential metabolites and pathway enrichment

**Fig 4:**
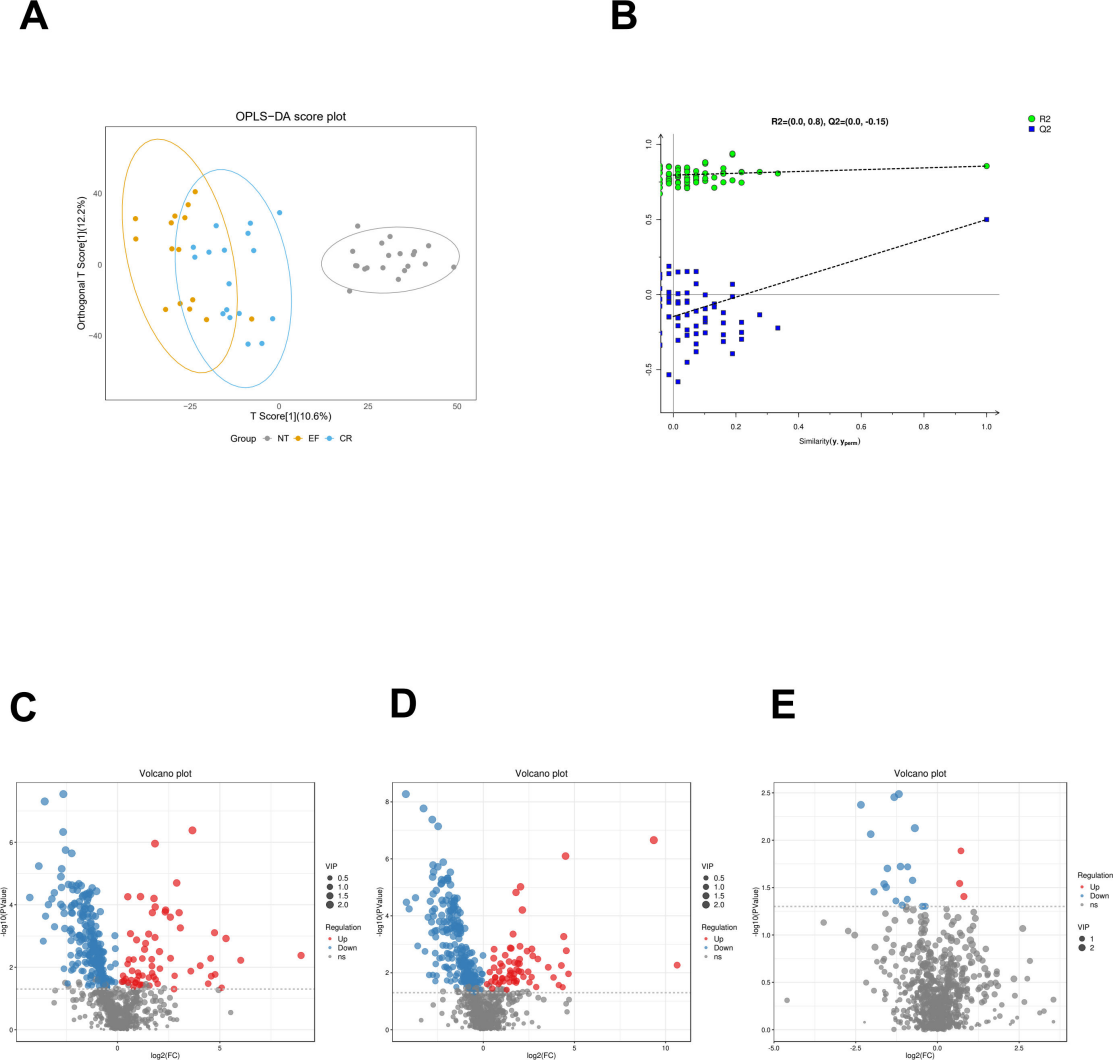
OPLS-DA score plot (**A**); OPLS-DA permutation test plot (**B**). The Y-axis shows R2Y and Q2, and the X-axis shows the correlation of observed and permuted data. The two points on the right side represent R2Y and Q2 of the observed data. Other points on the left side represent R2Ys and Q2s of permuted data; volcano plot with VIP scores for differential metabolites in NT vs EF (**C**), NT vs CR (**D**), and EF vs CR (**E**).

The OPLS-DA result was similar to previous analysis, which indicates that the NT group could be distinguished from the other two groups (EF and CR); however, the difference between the EF and CR groups was not significant (Fig. 4A). The 100 random permutation tests were performed to evaluate whether there is overfitting in the OPLS-DA models by R package ropls. The criteria for OPLS-DA model validity are as follows: (i) all Q2 values on the permuted data to the left are lower than the Q2 value on the actual data to the right, and (ii) the regression line (line joining the point of observed Q2 to the centroid of a cluster of permuted Q2 values) has a negative value of intercept on the Y-axis. The permutation test result showed that there was no overfitting in our OPLS-DA models (Fig. 4B). The differential metabolites distribution with VIP scores can also be viewed in Fig. 4C through E.

**Fig 5:**
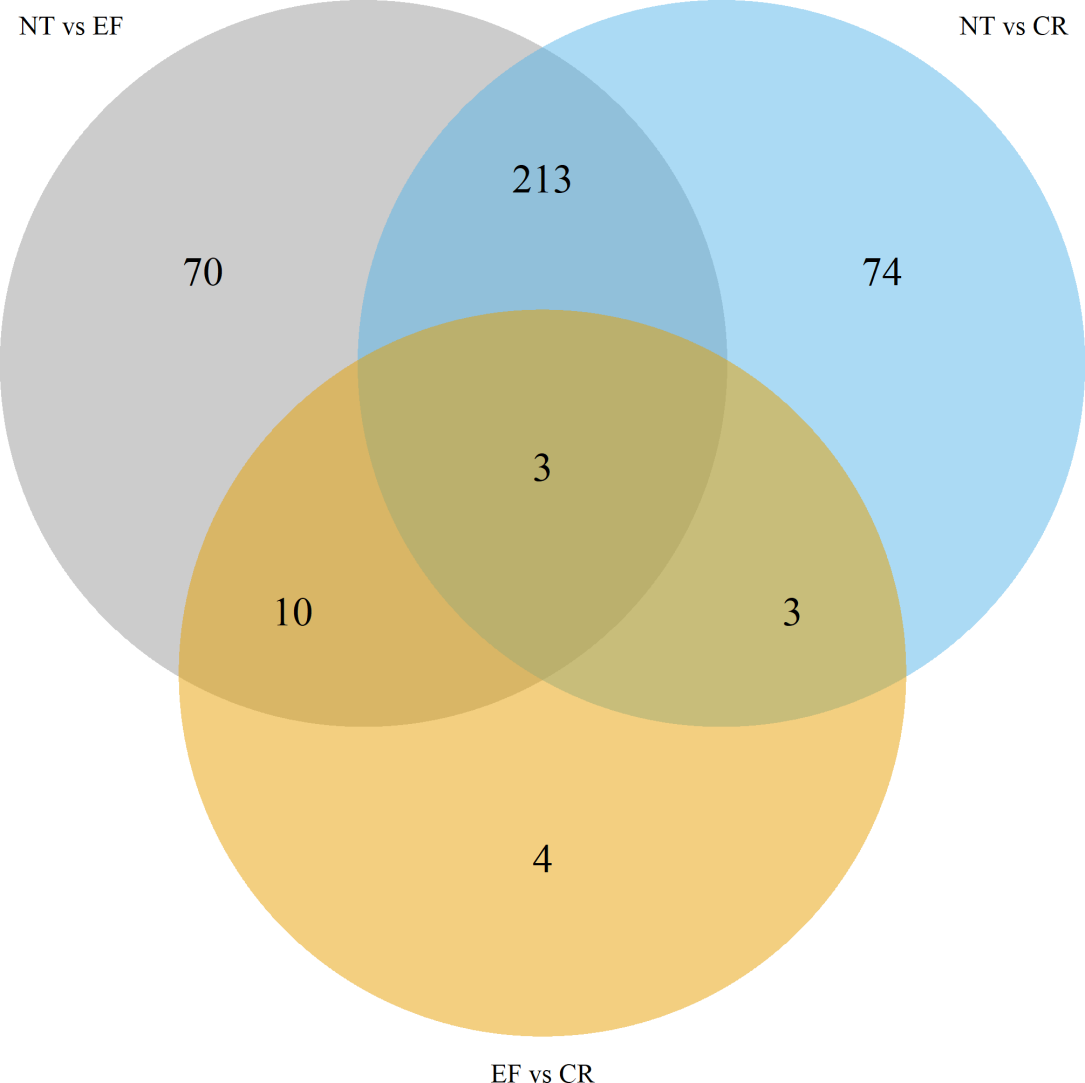
Venn plot differential metabolites among the three groups in pairwise comparisons.

More detailed information on differential metabolites among the three groups in pairwise comparisons is provided in Fig. 5; Tables 4 to 6. The comparative analysis of metabolites between the NT and EF groups, as well as between the NT and CR groups, revealed a substantial level of similarity, with a shared pool of 216 compounds. Also, within these groups, there were three specific metabolites, namely, 8,11,14-eicosatrienoic acid, cathasterone, and guanine, which exhibited statistically significant differences in all pairwise comparisons.

**TABLE 4:** Differential metabolites between NT and EF (top 10, sorted by increasing *P* values) log_2_ fold change (log_2_FC) > 0, indicating increased abundance in the EF group, and log_2_FC < 0, indicating decreased abundance in the EF group

Name	Log_2_FC	VIP	*P* value
2-Isopropyl-3-oxosuccinate	−2.65	2.16247424	2.87534E-08
Acetaminophen	−3.56	2.152918719	4.94058E-08
4-Methylbenzyl alcohol	3.66	2.054737216	4.16495E-07
8,11,14-Eicosatrienoic acid	−2.66	2.026486598	4.69456E-07
Acetoacetic acid	1.83	2.000744685	1.10506E-06
1,8-Diazacyclotetradecane-2,9-dione	−2.54	1.918850478	1.78313E-06
Capsanthin	−2.24	1.987099741	2.26149E-06
Isochorismate	−3.85	1.908520449	5.78193E-06
N6-Acetyl-LL-2,6-diaminoheptanedioate	−2.73	1.923223895	7.1088E-06
All-trans-Retinoic acid	2.18	2.034792441	1.389E-05

**TABLE 5:** Differential metabolites between CR and NT (top 10, sorted by increasing *P* values) log_2_ fold change (log_2_FC) > 0, indicating increased abundance in the CR group, and log_2_FC < 0, indicating decreased abundance in the CR group

Name	Log_2_FC	VIP	*P* value
2,3-Butanediol	−4.25	2.248485748	5.32329E-09
*cis*-4-Hydroxy-D-proline	−3.28	2.218666292	1.70087E-08
19(S)-HETE	−2.8	2.134052426	4.18841E-08
2-Isopropyl-3-oxosuccinate	−2.48	2.093815335	7.22847E-08
Guanine	9.35	2.319957223	2.20235E-07
Palmitoyl-L-carnitine	4.51	1.981911508	7.99633E-07
Caryophyllene epoxide	−2.21	2.006216881	1.3112E-06
Zerumbone	−2.76	1.98119827	1.65038E-06
Polygodial	−2.5	1.965511356	1.90219E-06
N6-Acetyl-LL-2,6-diaminoheptanedioate	−2.73	1.922722636	2.86672E-06

**TABLE 6:** Differential metabolites between CR and EF (top 10, sorted by increasing *P* values) log_2_ fold change (log_2_FC) > 0, indicating increased abundance in the CR group, and log_2_FC < 0, indicating decreased abundance in the CR group

Name	Log_2_FC	VIP	*P* value
Pseudouridine	−1.18	2.813885333	0.003258642
Methyl hexadecanoic acid	−1.32	2.642608281	0.003512261
8,11,14-Eicosatrienoic acid	−2.34	2.602505049	0.004231731
*trans*-Ferulic acid	−0.69	2.896672446	0.007453856
L-Erythrulose	−2.04	2.508040375	0.008631036
L-Dopa	0.72	1.689693692	0.012956558
Palmitic acid	−1.13	2.422406384	0.018954908
Cathasterone	−0.91	1.879020243	0.01908985
Cadaverine	−1.53	2.393410925	0.019845041
Trigonelline	−0.76	2.190190931	0.026523388

**Fig 6:**
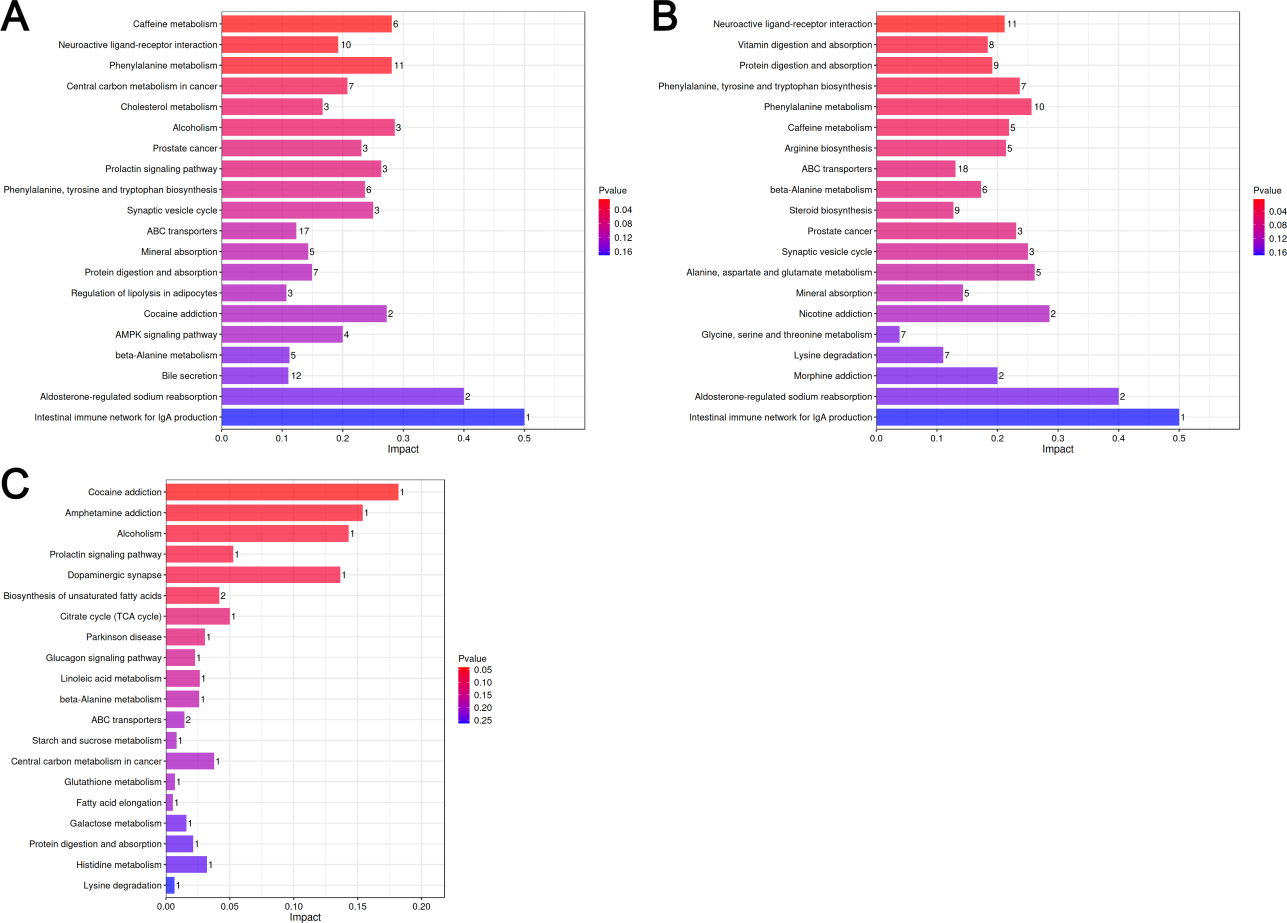
KEGG pathway enrichment analysis of differential metabolites in NT vs EF (**A**), NT vs CR (**B**), and EF vs CR (**C**).

Multiple consistent pathways were observed in the comparison between the NT and EF groups (Fig. 6A) and the NT and CR groups (Fig. 6B). These pathways include “ABC transporters,” “aldosterone-regulated sodium reabsorption,” “caffeine metabolism,” “mineral absorption,” “neuroactive ligand–receptor interaction,” “phenylalanine, tyrosine, and tryptophan biosynthesis,” “protein digestion and absorption,” “prostate cancer,” “synaptic vesicle cycle,” and “intestinal immune network for IgA production.” Among these pathways, the one with the highest impact score was “intestinal immune network for IgA production,” and the associated differential metabolite was identified as all-trans retinoic acid (ATRA), also known as Tretinoin.

In the KEGG results of EF vs CR (Fig. 6C), several pathways were also identified; however, apart from “biosynthesis of unsaturated fatty acids” and “ABC transporters,” all other pathways only contained one differential metabolite. Two metabolites, palmitic acid and 8,11,14-eicosatrienoic acid, were enriched in the “biosynthesis of unsaturated fatty acids” pathway. Both palmitic acid and 8,11,14-eicosatrienoic acid showed decreased abundance in the CR lung transplant rejection group compared to the EF group, indicating that the synthesis of unsaturated fatty acids might be inhibited during lung transplant rejection. We also found that palmitic acid was a differential metabolite in the NT vs EF comparison, with a significant increase in abundance in the EF group.

### Microbiome and metabolome correlation

**Fig 7:**
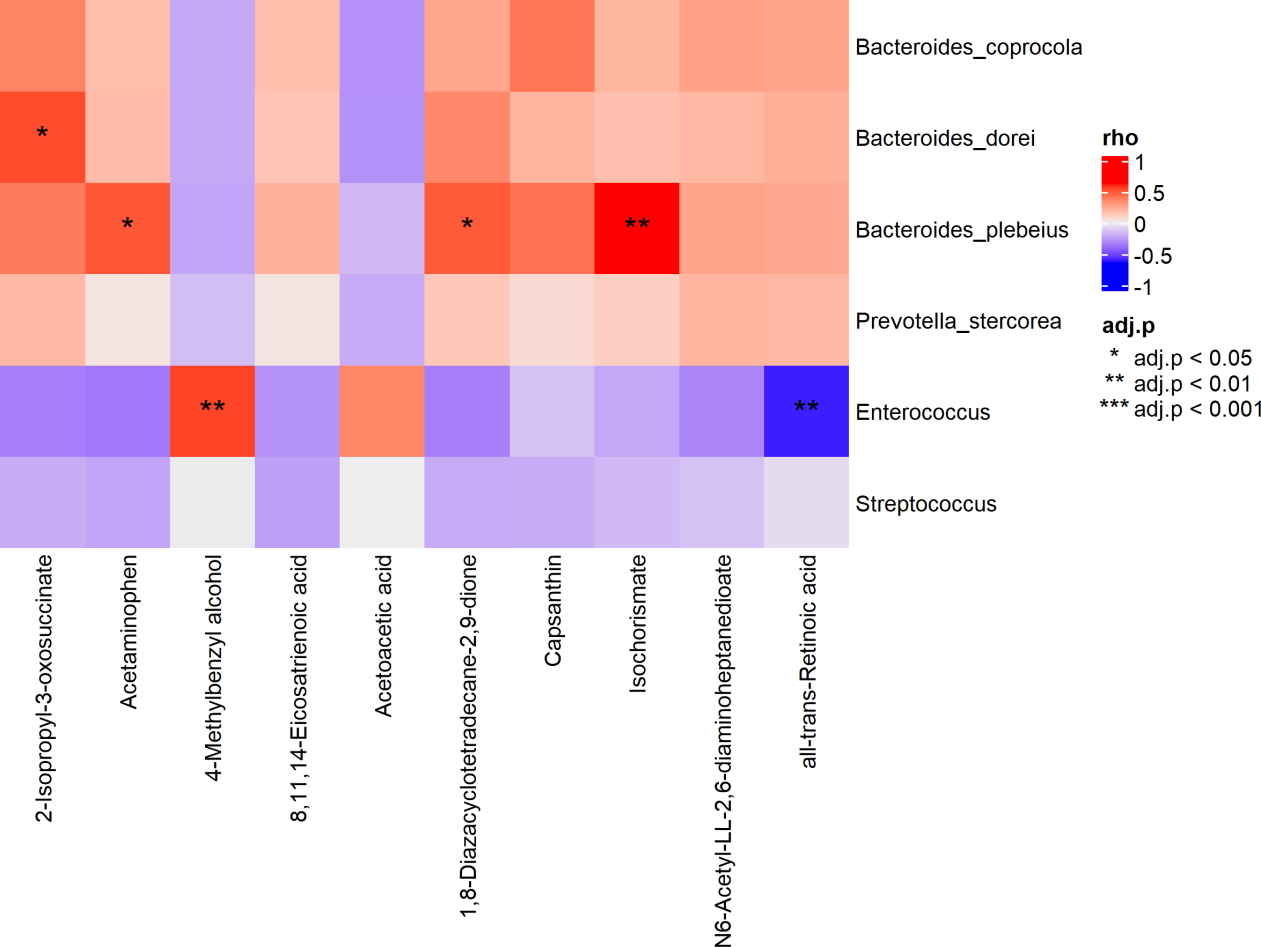
Spearman correlation heatmap of differential microbial species and the top 10 differential metabolites in the lung transplant vs no transplant group; *adjusted *P* value < 0.05, ** <0.01, *** <0.001.

In the lung transplant vs no transplant group, we examined the Spearman correlation between differential microbial species (if not found on species level, we chose genus level) and the top 10 significantly differential metabolites in the lung transplant vs no transplant group (Fig. 7). The significant correlations (adjusted *P* value < 0.05) are presented as follows:

1. *Bacteroides dorei*: positively correlated with 2-isopropyl-3-oxosuccinate.
2. *Enterococcus*: positively correlated with 4-methylbenzyl alcohol and negatively correlated with all-trans retinoic acid (ATRA) Fig. 7.

In the comparison between the EF and CR groups, we also performed Spearman correlation analysis between the only one differential bacterial genera *Ruminococcus gnavus* group and differential metabolites; however, no significant correlation could be observed (adjusted *P* value > 0.05) Fig. 7.

## DISCUSSION

Lung transplantation has emerged as a transformative treatment modality for individuals with end-stage lung diseases, offering renewed hope and improved quality of life. Despite the significant advancements, the intricate interplay between the gut microbiota and lung transplantation outcomes remains a relatively unexplored area of research. To address this knowledge gap, we conducted a comprehensive study employing 16S metagenomics sequencing to analyze changes in the gut microbiome, along with untargeted metabolic analysis to investigate alterations in the metabolome, following lung transplantation. By categorizing participants into three distinct groups (NT, EF, and CR), the present study aims to reveal the previously unexplored relationship between the gut microbiota and lung transplantation, offering valuable insights into potential therapeutic avenues and personalized interventions.

The presence of *Enterococcus* and *Streptococcus* as dominant genera in the lung transplant groups (both EF and CR) compared to the NT group provided insight into the microbial composition following lung transplantation. More specifically, certain species within these genera, such as *Enterococcus faecalis*, *Enterococcus faecium*, and *Streptococcus pneumoniae*, have been documented as frequent sources of infection following solid organ transplantation (22, 23). Conversely, the reduced presence of *Faecalibacterium*, a genus recognized for its ability to metabolize glucose, in patients undergoing lung transplantation might partially account for the elevated occurrence of diabetes mellitus during the post-transplantation recovery phase (24–26). We also observed a notable decrease in various species within the genus “*Bacteroides*,” including *Bacteroides coprocola*, *Bacteroides dorei*, and *Bacteroides plebeius*, in the lung transplantation group patients. The genus “*Bacteroides*” can synthesize short-chain fatty acids, especially butyric acid, which holds a pivotal function in augmenting the immune response of the host organism (27, 28).

The microbial composition of the two lung transplant groups EF and CR exhibited remarkable similarity, with the exception of the *Ruminococcus gnavus* group, which was found to be significantly more abundant in the EF group. *Ruminococcus gnavus* group is related to an inflammatory bowel disease called Crohn’s disease, and it can secrete an inflammatory polysaccharide that stimulates dendritic cells to produce inflammatory factors such as TNFα (29). The relationship between the gut microbiota *Ruminococcus gnavus* group and organ transplantation has been rarely reported. Only one singular study conducted in Tunisia provided evidence that the gut abundance of the *Ruminococcus gnavus* group was notably diminished in kidney transplant recipients exhibiting symptoms of obesity, diabetes, hypertension, or dyslipidemia, in comparison to those recipients who did not present any of these symptoms (30). Based on these findings, we predicted that *Ruminococcus gnavus* group might be associated with the prognosis of lung transplant recipients.

By employing a combination of differential metabolite analysis and KEGG pathway enrichment analysis, we identified a significant decrease in ATRA levels and inhibition of IgA production in lung transplant recipients. IgA is a crucial component of the mucosal immune system and plays a pivotal role in defending against pathogens at mucosal surfaces (31). The secretion of IgA is known to be enhanced by retinoic acid (RA), with ATRA being one of the most common forms of RA (32, 33). The inhibition in IgA production pathway observed in our study has important implications for the protective barrier function of the respiratory tract in lung transplant recipients. This compromised barrier function renders these individuals more susceptible to infections, which can have detrimental effects on their overall health and post-transplant outcomes. Our findings align with previous reports that have also highlighted the common occurrence of decreased levels of secretory IgA in lung transplant recipients (34, 35). In addition, we also found that the lung transplant dominant genus *Enterococcus* was negatively associated with ATRA level, which means that *Enterococcus* genus might be involved in the inhibition of IgA production in lung transplant recipients. Although there are no relevant studies on ATRA, one compound named CD437, which is in the retinoid family with ATRA, has been reported to have a significant inhibitory effect on *Enterococcus faecium* and *Enterococcus faecalis* in *Enterococcus* genus (36). These findings underscore the importance of monitoring and addressing the levels of secretory IgA, *Enterococcus* genus in the post-transplant period. Strategies aimed at enhancing IgA synthesis or supplementing with exogenous IgA may hold promise in reducing the risk of postoperative infections in lung transplant recipients.

As previously mentioned, the majority of the analysis results in the present study demonstrate minimal differences in the microbiota and metabolomic profiles between successful lung transplant recipients and those experiencing rejection. Furthermore, there is no significant correlation between the differential microbiota and differential metabolites between the two groups of patients. Therefore, we speculate that the success or failure (rejection) of lung transplantation is not significantly associated with the gut microbiota and its metabolome.

It is also important to acknowledge the limitations of the present study. First, we would like to clarify that due to the limited scope of our study and the available patient data, we were unable to include all potential confounders in our analysis, for example, gender, age, and allergies. However, we recognize the importance of considering these parameters and their potential role as confounders in the context of gut microbiota research. Secondly, species classification shows low sensitivity (~22%) when sequencing only the V4–V5 region of the 16S rRNA (37), resulting in many analyses in this study only being carried out at the genus level. Hence, our future research will be combined with shotgun metagenomic sequencing as much as possible, to identify more species-level microorganisms. Moreover, despite our efforts to correlate microbiota with metabolites through Spearman correlation analysis, further animal clinical trials should be conducted to establish direct associations. Additionally, untargeted metabolomic analysis relies on existing databases, which might overlook certain key metabolites. Although microbial distribution in the human body exhibits “organ specificity,” there are microbes that simultaneously inhabit the gut and lungs. Hence, further animal clinical trials are needed to determine whether the primary influence is exerted by gut microbiota or lung microbiota, and to elucidate the underlying mechanisms.

### Conclusion

This comprehensive study investigating the relationship between the gut microbiota, metabolome, and lung transplantation outcomes has provided valuable insights into potential therapeutic avenues and personalized interventions. Although we observed distinct microbial compositions and metabolic alterations following lung transplantation, the success or chronic rejection of the transplantation itself does not appear to be significantly associated with the gut microbiota and its metabolome. Our findings highlight the importance of monitoring and addressing specific microorganisms and metabolites, such as *Enterococcus* genus, ATRA, and secretory IgA, in the post-transplant period to reduce the risk of infections and improve patient outcomes. Further research, including animal clinical trials, is warranted to establish direct associations and elucidate the underlying mechanisms of these observations.

## Data Availability

Raw 16S amplicon metagenomic sequencing data are available at the National Center for Biotechnology Information (NCBI), under BioProject PRJNA1049134 with SRA accession numbers from SRX22793616 to SRX22793667.
